# Publisher Correction to: Fungal Biology and Biotechnology, volume 6

**DOI:** 10.1186/s40694-019-0074-9

**Published:** 2019-07-31

**Authors:** Fungal Biology and Biotechnology

**Affiliations:** London, UK

## Publisher Correction to: Fungal Biol Biotechnol (2019) 6:8 10.1186/s40694-019-0071-z, Fungal Biol Biotechnol (2019) 6:9 10.1186/s40694-019-0072-y

An error occurred during the publication of a number of articles in *Fungal Biology and Biotechnology*. Several articles were published in volume 6 with a duplicate citation number.

In this correction article the old and new citation metadata are published in Table 1.

**Table 1:** Overview of incorrect and correct citation metadata

DOI	Incorrect citation number	Correct citation number
10.1186/s40694-019-0071-z	1	8
10.1186/s40694-019-0072-y	2	9

The original articles have been updated. The publisher apologizes for the inconvenience caused to our authors and readers.

